# Plant Growth-Promoting Soil Bacteria: Nitrogen Fixation, Phosphate Solubilization, Siderophore Production, and Other Biological Activities

**DOI:** 10.3390/plants12244074

**Published:** 2023-12-05

**Authors:** Anna M. Timofeeva, Maria R. Galyamova, Sergey E. Sedykh

**Affiliations:** 1 Institute of Chemical Biology and Fundamental Medicine, Siberian Branch of the Russian Academy of Sciences, 630090 Novosibirsk, Russia; anna.m.timofeeva@gmail.com; 2 Faculty of Natural Sciences, Novosibirsk State University, 630090 Novosibirsk, Russia; mgalyamova@gmail.com

**Keywords:** plant growth-promoting bacteria, PGPB, soil bacteria, rhizosphere bacteria, phosphate solubilization, biofertilizers, nitrogen fixation, phosphate fertilizers, siderophores, sustainable agriculture

## Abstract

This review covers the literature data on plant growth-promoting bacteria in soil, which can fix atmospheric nitrogen, solubilize phosphates, produce and secrete siderophores, and may exhibit several different behaviors simultaneously. We discuss perspectives for creating bacterial consortia and introducing them into the soil to increase crop productivity in agrosystems. The application of rhizosphere bacteria—which are capable of fixing nitrogen, solubilizing organic and inorganic phosphates, and secreting siderophores, as well as their consortia—has been demonstrated to meet the objectives of sustainable agriculture, such as increasing soil fertility and crop yields. The combining of plant growth-promoting bacteria with mineral fertilizers is a crucial trend that allows for a reduction in fertilizer use and is beneficial for crop production.

## 1. Introduction

In the second half of the twentieth century, the use of mineral fertilizers was one of the main factors contributing to crop production development. However, unbalanced fertilizer application has been reported to have a negative impact on crop production sustainability and environmental safety [1]. Another consequence is the loss of soil microbiome diversity, leading to reduced fertility [2,3]. A promising research avenue currently being explored is the use of bacteria to enhance soil fertility and stimulate crop yields [4]. Plant growth-promoting bacteria (PGPB) and their consortia [5] can naturally enhance plant growth, both directly and indirectly, by fixing atmospheric nitrogen [6], synthesizing plant hormones and siderophores [7], stimulating plant nutrient uptake [8], and suppressing pests and pathogens [9,10]. Of particular significance is the fact that such bacteria interact with plant roots and increase resistance to abiotic stresses [11].

This review provides a comprehensive account of the various ways in which soil bacteria can be used to promote plant growth. We describe the nitrogen-fixing, phosphate-solubilizing, and siderophore-producing activities of soil microorganisms. Additionally, we discuss the creation of microbial consortia—including combining these with fertilizers—that can be introduced into soil to address the current challenges of sustainable agriculture.

## 2. Nitrogen-Fixing Bacteria

Bacteria that fix atmospheric nitrogen (N_2_) live in plant tissues (e.g., tubers and roots) and at the soil–rhizosphere interface, and can supply the significant amounts of mineral nitrogen required for plant growth [12,13]. Nitrogen fixation has been described for both symbiotic legume bacteria and non-symbiotic soil bacteria (heterotrophic or autotrophic) found in soil or water, or on stones or fallen leaves. Symbiotic bacteria of legume nodules are believed to be the most critical component of the biological fixation of atmospheric nitrogen [14]. Non-symbiotic nitrogen-fixing soil bacteria are significantly underrepresented in the literature; existing publications are mainly concerned with cereal crops such as maize, rice, and wheat [15,16,17]. Non-symbiotic nitrogen-fixing bacteria that increase the productivity of cereal crops have been described for the following genera: *Azospirillum*, *Azotobacter*, *Beijerinckia*, *Burkholderia*, *Clostridium*, *Gluconacetobacter*, *Herbaspirillum*, *Methanosarcina*, and *Paenibacillus* [15,18,19].

### Non-Symbiotic Nitrogen-Fixing Bacteria

Free-living nitrogen-fixing bacteria have been reported to use, rather than fix, nitrogen when mineral nitrogen is available in soil [20,21]. The basic enzyme of nitrogen fixation is nitrogenase. Its activity is sensitive to oxygen; requires metals that are part of the enzyme subunits (such as Fe, V, and Mo); depends on ATP and reduced coenzymes; and is low in the presence of mineral nitrogen. Free-living nitrogen-fixing bacteria can be obligate anaerobes, facultative anaerobes, or obligate aerobes found in different environments at different molecular oxygen concentrations. O_2_ can inhibit nitrogenase and suppress N_2_ fixation. Nitrogen-fixing bacteria can avoid the potentially negative effects of O_2_ by isolating the nitrogen fixation in space, e.g., by using structures such as heterocysts, where the oxygen concentration is kept low [22].

According to several reports, nitrogen-fixing soil bacteria supply a significant amount of the mineral nitrogen used in agriculture [23], making nitrogen fixation the second most important biogeochemical process after photosynthesis. This energy-dependent process requires sixteen ATP molecules to fix one atmospheric nitrogen molecule [6]. Soil bacteria can fix atmospheric nitrogen while exhibiting diazotrophic activity. However, they can also convert mineral nitrogen into NO, N_2_O, and N_2_, which is considered undesirable for agroecosystems, especially since N_2_O is a greenhouse gas. One strain of soil bacteria tends to contain different genes for nitrogen metabolism, with their expression largely dependent on mineral nitrogen availability in the environment. Variants of the soil bacterial consortia have been proposed, which can perform nitrogen fixation and contribute to nitrogen accumulation in the soils of agroecosystems [22].

All known forms of nitrogenase require Fe atoms, with most of them also containing metals such as Mo or V [24]. The *nifH* gene, encoding nitrogenase reductase [25] as well as several other genetic markers, is widely used to analyze the ability of bacteria to fix nitrogen, as well as the distribution of nitrogen-fixing agents in communities.

The availability of cellular ATP and soluble phosphates in the environment significantly influences the process of nitrogen fixation. Additionally, this process depends on the availability of iron ions in the environment and cellular Fe-containing cofactors and enzymes. Nitrogen fixation, in turn, provides the nitrogen compounds necessary for metabolic processes. Given the above, it is beyond doubt that the processes of nitrogen fixation, phosphate solubilization, and siderophore synthesis are interrelated. The synergetic effect of the simultaneous introduction of a consortium of bacteria exhibiting these activities individually, or several activities simultaneously, into the soil can stimulate plant growth and development, meeting the goals and objectives of sustainable agriculture and contributing to the economical and rational use of fertilizers.

## 3. Phosphate-Solubilizing Microorganisms

Phosphate-solubilizing bacteria [26] and mycorrhizal fungi [27] are known to increase the bioavailability of phosphorus from soil to plants [28]. They solubilize inorganic phosphates and mineralize insoluble organic forms of phosphorus [29]. Microorganisms capable of solubilizing phosphorus have been considered in a previous review [8], which describes the phosphate-solubilizing activity of bacteria belonging to the following genera: *Aeromonas*, *Agrobacterium*, *Azotobacter*, *Bacillus*, *Bradyrhizobium*, *Burkholderia*, *Cyanobacteria*, *Enterobacter*, *Erwinia*, *Kushneria*, *Micrococcus*, *Paenibacillus*, *Pseudomonas*, *Rhizobium*, *Rhodococcus*, *Salmonella*, *Serratia*, *Serratia*, *Sinomonas*, and *Thiobacillus*. Additionally, consideration is given to fungi belonging to *Achrothcium*, *Alternaria*, *Arthrobotrys*, *Aspergillus*, *Cephalosporium*, *Chaetomium*, *Cladosporium*, *Cunninghamella*, *Curvularia*, *Fusarium*, *Glomus*, *Helminthosporium*, *Micromonospora*, *Phenomiocenspora*, *Phenomiocenspora*, *Phenomycylum*, *Populospora*, *Pythium*, *Rhizoctonia*, *Rhizopus*, *Saccharomyces*, *Schizosaccharomyces*, *Schwanniomyces*, *Sclerotium*, *Torula*, *Trichoderma*, and *Yarrowia*. Among all phosphate-solubilizing microorganisms, bacteria significantly predominate, accounting for up to 50%, while fungi cover up to 0.5%. Most phosphate-solubilizing microorganisms are found in the rhizosphere [30].

### 3.1. Solubilization of Inorganic Phosphorus Compounds

Phosphorus compounds in soil can be both inorganic and organic. The following inorganic phosphate compounds have been described: apatite, strengite, and variscite; in addition to secondary minerals, such as ferric, aluminum, and calcium phosphates [31]. It has been established through various studies that the primary mechanism responsible for the mineral phosphate solubilization exhibited by bacteria is the secretion of organic acids [28,31,32]. Soil bacteria have been reported to secrete organic acids, stimulating phosphate solubilization by acidifying the environment and by chelating metal ions from the corresponding inorganic compounds [32,33].

The solubilization efficiency also significantly depends on the strength and chemical structure of organic acids. For example, carboxylic acids containing a single carboxyl group are known to be less efficient than dicarboxylic or tricarboxylic acids. It has also been demonstrated that aromatic organic acids are less active than the corresponding aliphatic analogs. The methods of mass spectrometry analysis, gas chromatography, and high-performance liquid chromatography [8] have proven that the organic acids secreted by rhizosphere bacteria in soil are involved in phosphate solubilization. These include acetic, adipic, butyric, citric, fumaric, glutaric, glycolic, glyconic, glyoxalic, 2-ketogluconic, lactic, malic, malonic, oxalic, propionic, succinic, and tartaric acids [30], with gluconic and 2-ketogluconic acids apparently being the most important.

The secretion of organic acids by bacterial cells is associated with several metabolic pathways, such as the direct oxidation of low molecular weight precursors in the periplasm [34], and intracellular phosphorylation [35].

### 3.2. Mineralization of Organic Phosphorus Compounds

Soil’s organic phosphorus compounds can account for up to 30–50% of soil phosphorus. Organic phosphorus compounds are mainly found in the form of phytate (inositol phosphate). The other organic soil phosphates described in the scientific literature include nucleic acids; mono-, di-, and tri-esters; and phospholipids [26]. Xenobiotic phosphonates are organic phosphorus compounds that can also be found in high concentrations in soil. These include antibiotics, detergents, pesticides, flame retardants, and other compounds. All the molecules mentioned above must be converted into soluble ionic phosphate forms before being assimilated by plant roots [36].

Organic compounds are metabolized by enzymes secreted into the environment by phosphate-solubilizing bacteria. Nonspecific acidic phosphatases that cleave phosphate from the ester or phosphoanhydride bond are represented by phosphomonoesterases: alkaline and acidic phosphatases [37,38]. The phytase enzyme has been demonstrated to cleave phytates [39]. Acid phosphatase activity has been observed in *Pseudomonas fluorescens* [40,41], *Burkholderia cepacia* [42], *Enterobacter aerogenes*, *Enterobacter cloacae*, *Citrobacter freundi*, *Proteus mirabalis* and *Serratia marcenscens* [43], and *Klebsiella aerogenes* [44]. Additionally, phytase activity has been demonstrated for *Bacillus subtilis*, *Pseudomonas putida*, and *Pseudomonas mendocina* [45]. Interestingly, the secretion of phosphatases by soil bacteria significantly depends on both the free phosphate already available in the soil and the availability of inorganic nitrogen [46,47], indicating a close relationship between nitrogen fixation and phosphate solubilization processes, and possible synergism of bacterial consortia combining these activities.

## 4. Siderophore-Secreting Microorganisms

Fe^2+^ ions are involved in various biochemical intracellular processes in plants, such as atmospheric nitrogen fixation (see Section 1) and photosynthesis [48]. Although iron is one of the most common elements in the Earth’s crust, its availability for plant root uptake is extremely low due to the rapid oxidation of Fe^2+^ ions to Fe^3+^ ions in the environment, making them insoluble [49].

Bacteria and other microorganisms are known to secrete siderophores—organic compounds with a molecular mass of up to 1.5 kDa and a high affinity for Fe^3+^ [50,51,52]. Siderophores secreted by rhizosphere microorganisms promote the conversion of Fe^2+^ ions into a form available to plant roots [53,54], favoring plant growth and development.

Siderophore-producing bacteria have been described in twenty genera [7], including *Azospirillum*, *Azotobacter*, *Bacillus*, *Dickeya*, *Klebsiella*, *Nocardia*, *Paenibacillus*, *Pantoea*, *Pseudomonas*, *Serratia*, *Streptomyces*, and others. A number of genera from this list are also known to contain atmospheric nitrogen-fixing and phosphate-solubilizing bacteria. Some bacteria have been reported to exhibit multiple activities that promote plant growth and development.

It should be noted that siderophores are not secreted only by soil bacteria. They can also be produced by human pathogenic bacteria [55], fungi [56,57], and oomycetes [58].

### 4.1. Siderophores Produced by Soil Bacteria

The siderophore structure comprises an iron atom surrounded and coordinated by oxygen atoms. The octahedral configuration that is most common in bacterial siderophores contributes to the stabilization of Fe^3+^ ions [59]. Fe^3+^ ions can be coordinated by various functional structural groups, with the most common being catecholates, hydroxamates, α-hydroxycarboxylates, and combinations of nitrogen-containing heterocycles, phenolates, and carboxylates [60]. Siderophores are classified by structure and chemical nature as catecholate, hydroxamate, carboxylate, and phenolate siderophores [61,62]. Some siderophores are classified as mixed, due to their structure corresponding to two or three classes simultaneously.

The structure of hydroxamate siderophores includes a functional group C(=O)N-(OH)R, with R being an amino acid or its derivative. This group contains two oxygen atoms forming a ligand with Fe^2+^ ions [61]. Hydroxamate schizokinen siderophores have been described in *Bacillus megaterium* [63]. The *Rhizobium leguminosarum* strain has also been found to produce a schizokinen siderophore [64]. Other hydroxamate siderophores have been described in *Pantoea vagans* [65], *Rhizobium meliloti* [66], *R. leguminosarum*, and *R. phaseoli* [67,68].

Catecholate siderophores contain Fe^3+^ ions bound to catecholate or hydroxyl groups. The structure of the resulting complex is also octahedral [69]. Catecholate siderophores are most commonly derived from either 2,3-dihydroxybenzoic acid or salicylic acid [70]. Catecholate siderophores have been described in bacteria of the genus *Azospirillum*, including *A. brasilense* [71], *A. lipoferum* [72], and *A. vinelandii*, which are known to produce four different catecholate siderophores [61,73,74]. Other siderophores have been described in *Rhizobium leguminosarum* [64], *R. radiobacter* (agrobactin) [75,76], *Bacillus subtilis* (itoic acid) [77], and *B. thuringiensis* (bacillibactin) [78].

Siderophores containing several Fe^2+^ chelating groups are categorized as the mixed type. *Pseudomonas* strains secreting pyoverdines have been described [79,80], with their structure comprising a quinoline chromophore, a peptide, and a dicarboxylic acid or its amide attached to the chromophore [81].

For various strains of *Pseudomonas fluorescens*, several pyoverdins [82], enantio-pyochelin [83], quinolobactin [84], ornichorrugatin, and prepseudomonin have been described [85]. *Pantoea eucalypti* has been shown to secrete pyoverdine- and pyochelin-like siderophores in the alkaline medium [86]. The pyoverdine siderophore azotobactin has been described in the *A. vinelandii* strain [87].

It is noteworthy that Fe ions possess a higher affinity for bacterial siderophores compared to fungal siderophores, providing the former with antifungal properties. To date, the antifungal properties of the pyoverdine of *Pseudomonas* sp. have been investigated most thoroughly [88]. In summary, not only can pyoverdines favorably affect plant growth, but they can also exhibit antifungal properties and control relevant phytopathogens [89].

### 4.2. Life Cycle of Siderophores

Siderophores are synthesized in the cytoplasm and peroxisomes [49,90]. Next, siderophore molecules are secreted into the extracellular space. Following the binding of siderophores to Fe^3+^ ions, the complexes of iron ions and siderophores are transported into the periplasm by specific receptors. This transport mechanism—as opposed to a concentration gradient—is energy dependent, further confirming that the processes of ATP synthesis, atmospheric nitrogen fixation, and phosphate solubilization in the bacterial cell are interrelated.

The availability of iron ions for intracellular processes is due to two mechanisms: the reduction of Fe^3+^ ions to Fe^2+^ ions and the dissociation of the iron–siderophore complex, or the binding of ions to a higher-affinity acceptor [59]. Siderophore-interacting proteins, siderophore reductase, and enzymes that hydrolyze iron–siderophore complexes have been characterized [49]. It is apparent that the reduction of an iron ion in the siderophore complex is much more advantageous for the cell, allowing the siderophore molecule to be reused, which is unfeasible when siderophores are hydrolyzed by hydrolases.

Siderophores secreted by rhizosphere bacteria provide iron to plant roots and stimulate plant growth whenever the bioavailability of iron ions is low. Two mechanisms by which plants obtain iron ions from siderophores secreted by microorganisms have been described. The first mechanism involves the transport of siderophore complexes with iron ions to the apoplast, leading to siderophore reduction and Fe^2+^ ion delivery to the root. The second mechanism allows iron ions to be exchanged between bacterial siderophores and plant siderophores [91].

## 5. Isolation and Biochemical Analysis of Soil PGPB

**Figure 1:**
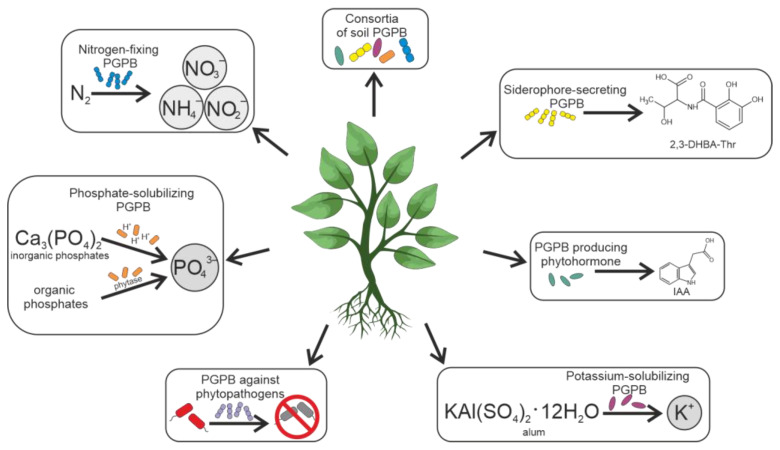
Different soil PGPB influence plant growth and development in various beneficial ways. These include fixation of atmospheric nitrogen, solubilization of phosphates and potassium, secretion of siderophores and phytohormones, and activity against plant pathogens. Moreover, bacterial consortia provide cumulative effects, favoring various aspects of plant viability.

Since it is impractical to conduct in vivo experiments with numerous bacterial isolates, the procedure of soil PGPB analysis is first directed toward the analysis of useful traits. Thus, PGPB are inoculated into plants, and the strains showing the best results are chosen, as described in [92]. Almost all methods for testing bacterial activity involve the primary isolation of pure cultures of microorganisms followed by inoculation into different media, with the corresponding activity assessed by growth or changes in color/transparency around the colony. Figure 1 illustrates the beneficial effects of soil PGPB on the soil’s biological activities towards promoting plant growth and development.

### 5.1. Isolation of Nitrogen-Fixing Bacteria

A 10 g soil sample is usually used to isolate soil bacteria. The procedure involves sample homogenization with 90 mL of sterile water, and using serial dilutions (1:10) up to 10^−6^. One milliliter of the resulting solution is applied to a Petri dish containing nutrient agar and incubated for 2–5 days at 28 °C [93]. After incubation, colonies of soil bacteria are dissolved in sterile saline.

To isolate diazotrophic (nitrogen-fixing) bacteria, diluted soil bacteria samples are inoculated into nitrogen-free semisolid media (Ashby’s Mannitol Agar or various other compositions) and incubated for up to seven days at 28 °C. After the incubation, samples in Petri dishes—with the media covered with colorless or colored colonies, plaque, or film—are considered positive for the presence of nitrogen-fixing bacteria. A single colony or bacterial biomass is taken from the surface of each culture medium using a sterile loop and used to inoculate fresh N-free semisolid media of the same type. This step is usually repeated two or three times to ensure that the isolates grow in a nitrogen-free culture medium. At each stage, a microscopic or molecular biological (PCR, 16S sequencing) control is used to confirm that a pure strain of bacteria is transferred to the next Petri dish [94].

### 5.2. Phenotypic Characteristics and Genotyping of Bacteria

Bacterial colonies are characterized by their morphological characteristics, such as cell and colony shape, color, elevation and opacity, and Gram staining [95]. To identify the genus (and species) of the strains, sequencing of the 16S rRNA gene is commonly used. This gene is used due to specific characteristics such as size, ubiquity among bacteria, and low rate of evolution. Bacterial genomic DNA is isolated [96]. Partial amplification of the 16S rRNA gene for Sanger sequencing is carried out using 27F (5′-AGAGTTTGATCCTGGCTCAG-3′) and 1492R (5′-GGTTACCTTGTTACGACTT-3′) primers. Maximum likelihood phylogenetic reconstruction of 16S rRNA genes allows strain classification to the genus or species level.

### 5.3. Metabolic Characterization of Plant Growth Promotion

Metabolic characterization of a strain, in the sense of biochemical activities associated with plant growth promotion, involves growing bacteria on Petri dishes or 96-well plates with different media, or assessing the production of bioactive compounds secreted by PGPB through analytical methods.

#### 5.3.1. Solubilization of Phosphates and Potassium

Phosphate solubilizing activity can be determined by growing bacterial isolates in a modified agar medium. This medium contains a 2% (*w*/*v*) inorganic source of phosphate, i.e., tricalcium phosphate (Ca_3_(PO_4_)_2_), referred to as *Pi*. Alternatively, lecithin is used as the organic phosphate source, and this medium is called *Po*. The cultivation is conducted at a temperature of 30 °C for seven days. Clear zones forming around bacterial colonies indicate that specific bacterial isolates are solubilizing phosphate [93,94].

The ability of bacteria to solubilize potassium is assessed with agar containing mica powder (0.2%) as the single source of potassium. Bacterial cultures that form a clear zone are considered K solubilizers [97,98].

These methods make it possible to quantify the biochemical characteristics of microorganisms. The ability of isolates to dissolve phosphorus is assessed by measuring the translucent halo around the colony. The phosphate solubilization index (PSI) and potassium solubilization index (KSI) are determined by calculation, using the equation in which the diameter of the clear zone is added to the colony diameter and the sum is divided by the colony diameter [98,99].

#### 5.3.2. Siderophore Production

PGPB isolates are inoculated onto chromazurol S (CAS) agar plates to screen for siderophore-producing activity. The formation of an orange halo around the bacterial colony indicates the production of siderophores [100]. Solid agar medium CAS can also be used to qualitatively determine siderophore production. Chromazurol S is a complex compound containing iron ions. The siderophores are strong Fe-chelators, and the removal of iron from the complex changes its color from blue to orange.

Based on this method, a quantitative assessment of the concentration of siderophores in the supernatant can be performed. This can be done by mixing the supernatant with the CAS reagent (1:1), and, after 20 min, by measuring the optical density at 630 nm [101]. The chromazurol assay does not reveal the type of bacterial siderophores. However, there are at least three other identification methods. The Arnow test [102] allows the presence of catechol groups in siderophores to be identified. The Csáky assay [103] is used to identify the presence of hydroxamate-type siderophores. The Shenker test can detect carboxylate siderophores [104].

#### 5.3.3. Analysis of Phytohormone Production

Auxin, ethylene, abscisic acid, cytokinin, and gibberellin are prominent classes of phytohormones, with each playing a distinct role in plant growth and development. PGPB can supply plants with significant amounts of auxin, resulting in modifications to the development of root systems [103,104]. These changes occur through the synergistic action of exogenous and endogenous auxin [105]. In addition, enzymes produced by plant growth-promoting bacteria, such as 1-aminocyclopropane-1-carboxylate (ACC) deaminase, indirectly contribute to architectural and functional modifications of the root [106].

Among the phytohormones produced by PGPB, auxins and indolyl-3-acetic acid (IAA) are the most studied [107,108]. The main pathway of their biosynthesis is carried out through tryptophan as a precursor [109], and a number of papers show the importance of bacterial IAA in promoting plant growth [110]. Several methods are used to determine IAA. One method is the determination of IAA using gas–liquid chromatography with a mass-selective detector (emission 360 nm, excitation 282 nm). This method requires the use of expensive equipment and complex sample preparation. The second method for determining IAA is the interaction of IAA with Salkovsky’s reagent (2 mL of 0.5 M solution of FeCl_3_ in 100 mL of 37% HClO_4_), which leads to a colored compound. Inoculants are grown in R2A broth containing 2 mM L-tryptophan, an IAA precursor. The supernatant is mixed with Salkovsky’s reagent, and the concentration of IAA is assessed spectrophotometrically via the optical density of the colored product at 530 nm [111,112,113].

The enzyme 1-aminocyclopropane-1-carboxylate deaminase is responsible for ethylene synthesis and indirectly contributes to architectural and functional modifications of the root [114]. Quantitative analysis of deaminase activity is based on the ability of microorganisms to assimilate 1-aminocyclopropane-1-carboxylate as the sole nitrogen source [115]. Bacteria are spot-inoculated into nitrogen-free saline DF (Dworkin and Foster) media on plates supplemented with ACC, and incubated at 30 °C for 5 days. Growth of isolates on ACC-supplemented plates demonstrates deaminase activity.

### 5.4. Analysis of Antagonistic Activity against Phytopathogens and Competition between Strains

A test for antifungal activity is used to analyze the antagonistic activity of bacterial strains [116]. Bacterial strains are evenly distributed over a Petri dish containing a potato dextrose agar medium. The plates are incubated at 4 °C for 2 h to promote the diffusion of bacteria into the medium, and then a 6 mm diameter fungal disk sample is placed in the center of each Petri dish. The plates are incubated under dark conditions at a temperature of 25 °C for a period of seven days. Calculation of the percentage inhibition of radial growth involves subtracting the *Rs* value (radius of fungus in the presence of bacteria) from the *Rc* value (radius of fungus control) and dividing the remainder by *Rc*, according to the formula [116]. Inhibition of the growth of the phytopathogen indicates the biocontrol of the bacterial strain studied.

A cross-test is used to assess antagonistic activity between bacterial strains [113]. The first strain is applied to the agar medium in perpendicular streaks and grown at 30 °C for 3 days. The second strain is then applied with strokes at an angle of approximately 90°, extending outward from the emerging colonies of the first strain. Both strains of bacteria are incubated at 30 °C for another 3 days. Colony lines and zones of inhibition that arise at the intersection of strains are visualized. Strain compatibility is characterized by the absence of inhibition zones at the intersection of two colonies. The incompatibility is caused by competition between bacteria.

### 5.5. Analysis of Plant Growth Promotion

Sterile plant seeds are germinated and grown under sterile conditions at 22 °C with a photoperiod of 16 h of light and 8 h of darkness [117]. Five- to ten-day-old seedlings are inoculated with a bacterial culture, with uninoculated plants used as controls. After inoculation, photographs of the plants are taken every two days to determine the length of the primary root and the number and density of lateral roots (density is the number of lateral roots or the length of the primary root). The seedlings are weighed and dried at 80 °C in paper bags until a constant weight is reached. Wet and dry biomass are determined.

### 5.6. Bacterial Genes Promoting Plant Growth

Several bacterial genes are already known to be involved in the beneficial effects observed in plants, such as (i) genes involved in atmospheric nitrogen fixation (*Nif* genes, which encode the nitrogenase complex and other regulatory proteins); (ii) genes responsible for the formation of legume nodules (*nod*); (iii) genes controlling pathogens (chi genes, which produce chitinases, and *sfp* genes, which produce surfactins); (iv) genes involved in the production of phytohormones (*acdS*, which encodes the production of ACC-deaminase, improving stress resistance by reducing ethylene levels in the plant, and *ipdC*/*ppdC*, which are involved in the production of indoleacetic acid); (v) genes involved in vitamin production (*pqq*, which encodes pyrroloquinoline quinone); and (vi) genes involved in nutrient mobilization (*bgl*/*ybg* genes, which are involved in phosphate solubilization, and rhb genes, which encode siderophore production).

The *nifHDK*, *nifDK*, *nifK*, and *nifD* genes predict nitrogen fixation ability in bacterial genomes [118]. Phytase is one of the main enzymes produced by phosphate-solubilizing bacteria during the mineralization of organic phosphorus. This enzyme releases phosphorus from organic materials in the soil, which is stored as phytate [39]. Gluconic acid secretion is the best-characterized phosphate solubilization mechanism, which is mediated by pyrroloquinoline quinone (PQQ)-dependent glucose dehydrogenase (GDH) [119]. Analysis of the genes (*pqqA*, *pqqE*, *pqqAD*, and *pqqD*) encoding the cofactor PQQ is used as a proxy for phosphate solubilization. Auxin, ethylene, abscisic acid, cytokinin, and gibberellin are well-known classes of phytohormones. PGPB can increase plant productivity by causing changes in plant roots via auxin production, which acts synergistically with endogenous auxin and alters the development of the plant root system [120]. Indole-3-pyruvate decarboxylase (IPDC) is a key enzyme in synthesizing heteroauxin, the most important auxin encoded by the *ipdC* gene.

Specific or universal primer pairs and Sanger sequencing, or NGS in the case of soil consortia, are used to analyze the genes mentioned above.

### 5.7. Environmental Factors That Can Affect PGPB and Increase or Decrease the Synthesis of Plant Growth-Promoting Substances

Various environmental factors are known to affect the production of plant growth-promoting substances by PGPB. These factors include interaction with other microorganisms in the soil, climate zone soil types, soil physicochemical properties, and environmental conditions [32]. For example, PGPB in soils exposed to extreme environmental conditions, such as saline–alkaline soils, soils with high nutrient deficiencies, or soils from extreme temperature environments, tend to solubilize more phosphate and potassium than bacteria in soils from more moderate conditions [28].

The PGPB activity is also affected by soil composition. The growth rate of microorganisms was found to be related to the concentration of soluble phosphate [121]. Additionally, the growth rate of the *Pseudomonas aeruginosa* strain was estimated to be 25 times greater under phosphate excess than under phosphate deficiency [122]. The PGPB-mediated mineral phosphate solubilization activity was found to be inhibited by soluble phosphate via a negative feedback mechanism [123]. In this way, the phosphate-solubilizing activity of bacteria is induced by low levels of exogenous soluble phosphate and inhibited by high levels of exogenous soluble phosphate [33].

Various abiotic stresses can result in higher production of various growth-promoting factors. For example, drought and/or salt stress can increase the sensitivity of many plants to various phytopathogens, frequently by reducing the ability of the plant to effectively attack the pathogen. Under unfavorable conditions, PGPB were found to increase the secretion of the phytohormones abscisic acid, salicylic acid, and ethylene, which are responsible for activating the signaling cascade of various genes involved in salt tolerance [124].

**Figure 2:**
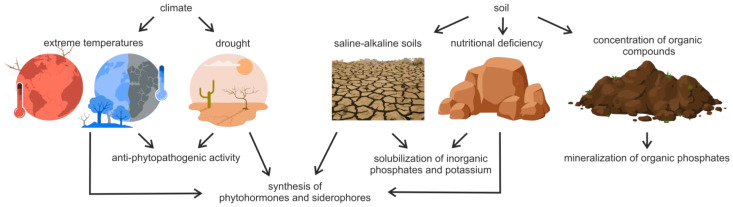
Environmental factors that affect the secretion of plant growth-favoring substances by soil bacteria.

Figure 2 provides a schematic representation of the environmental factors that affect PGPB and the secretion of substances that enhance plant growth.

## 6. Consortia of Soil PGPB

The soil microbiome is a complex community of millions of species with billions of possible interactions between them. Given that no soil bacterial species exist in isolation, these interactions result in new properties that are not characteristic of individual genera, species, and/or strains of bacteria [5]. Consortia formed through the interaction of several bacteria are promising for agricultural applications [125,126] compared to single-species inoculants [127]. However, it is essential for the species within a consortium not to be antagonistic to each other, and to be able to occupy a wider range of ecological niches than they would if they were isolated [128]. On the one hand, a more diverse microbial consortium may provide a greater number of functions beneficial to plants [129]. On the other hand, some strains of PGPB may simultaneously exhibit several activities favorable to plant growth. For example, several publications report that nitrogen-fixing bacteria exhibit phosphate-solubilizing and other activities. Alternatively, it is not known how actively a single bacterial strain can simultaneously exhibit several traits favorable for plant growth and development. Whatever the case, the application of microbial consortia can provide several plant-beneficial functions to the soil, to be performed simultaneously by different consortium members due to their mutual complementarity.

The application of a mixture of *Bacillus*, *Pseudomonas*, and *Streptomyces* isolated from soil and exhibiting phosphate-solubilizing activity has been shown to provide better results in accelerating the linear growth and root growth of plants compared to the use of individual strains. Another example is the inoculation of wheat seeds with *Pseudomonas fluorescens* Ms-01 and *Azosprillum brasilense* DSM1690 bacteria, which solubilize phosphates and secrete auxins that strongly increase root and shoot biomass compared to the application of the strains separately [130]. The consortium of *Bacillus firmus*, *Cellulosimicrobium cellulans*, and *Pseudomonas aeruginosa* has been demonstrated to increase the root length and linear growth of amaranth plants more effectively than when applying the strains one by one or in pairs [131]. In recent years, an increasing number of scientific papers have focused on the application of bacterial consortia with a positive effect on plant growth and development, corroborating their unequivocal relevance and significance.

### Combined Application of Bacteria and Phosphate Fertilizers

The coapplication of phosphate-solubilizing bacteria and some phosphate fertilizers has been demonstrated to produce a synergistic effect and increase agronomic efficiency, such as when using phosphorites (rock phosphates) [132,133,134]. Inoculation with phosphate-solubilizing bacteria has been found to significantly improve corn growth when applied with various inorganic and organic fertilizers: manure, poultry manure, superphosphate, and phosphate [135]. Inoculating wheat plants with *Pseudomonas* sp. or *Enterobacter* sp. and applying diammonium phosphate has been proven effective [131]. *Enterobacter* sp. has been reported to increase phosphorite solubilization from 17.5% [136] to 27% compared to the release from phosphorites (4%) alone [137]. A combination of a one-time inoculation of wheat plants with five different strains of *Pseudomonas* (*P. plecoglossicida*, *P. reinekei*, *P. koreensis*, *P. japonica*, and *P. frederiksbergensis*) and phosphate application was found to increase phosphate availability, nutrient availability, and chlorophyll content, and to improve the morphological characteristics associated with a higher phosphorus uptake [138]. A consortium of three strains, *P. corrugata*, *P. koreensis*, and *P. frederiksbergensis*, was found to enhance the plant growth of alfalfa (*M. truncatula*) when coapplied with phosphate fertilizers [139].

Phosphate-solubilizing soil bacteria are known not only to increase the efficiency of mineral phosphate fertilizers, but also to have beneficial effects when applied with organic fertilizers. For example, the application of a mixture of compost (biogas production residues) coupled with fossil phosphate-solubilizing bacteria *Bacillus* sp. was reported to enhance the availability of free phosphate in soil, increase the soil’s organic matter content, and boost the population of phosphate-solubilizing bacteria [140].

To summarize, the inoculation of crops with phosphate-solubilizing bacteria combined with mineral or organic fertilizers is a promising integrated strategy for increasing soluble phosphate availability, improving the agronomic efficiency of phosphate fertilizer usage, increasing soil fertility, and achieving sustainable agriculture goals.

## 7. Conclusions

Recent studies have shown that the application of PGPB as a biofertilizer is of great importance for the sustainable management of soil resources. The quality and nutritional value of agricultural products are of great significance to consumers. Further research in this area is necessary to provide farmers with timely information on the biological activity of PGPB. Atmospheric nitrogen fixation, phosphate solubilization, and increased iron bioavailability in soil due to soil bacteria all contribute to sustainable agricultural development, particularly by increasing soil fertility and crop yields. The usage of soil bacteria and their consortia for inoculation into plant seeds, as well as for coapplication with mineral fertilizers, may eventually contribute to developing a new generation of farming technologies with lower costs and reduced negative effects on the environment. Analyzing the molecular mechanisms of nitrogen fixation, phosphate solubilization, and siderophore synthesis, as well as identifying bacterial strains that can simultaneously exhibit several different activities, are necessary steps to create commercial bacterial preparations for enhancing plant growth in crops. One area that demands special consideration is the experimental testing of both individual strains and their consortia across various climatic zones, diverse soil types, and different crops. The authors of this review find such experimental research highly relevant due to the potential ineffectiveness of commercial preparations.

## Data Availability

Not applicable.
